# 30-day readmission, antibiotics costs and costs of delay to adequate treatment of Enterobacteriaceae UTI, pneumonia, and sepsis: a retrospective cohort study

**DOI:** 10.1186/s13756-017-0286-9

**Published:** 2017-12-06

**Authors:** Marya D. Zilberberg, Brian H. Nathanson, Kate Sulham, Weihong Fan, Andrew F. Shorr

**Affiliations:** 1 EviMed Research Group, LLC, PO Box 303, Goshen, MA 01032 USA; 2 OptiStatim, LLC, Longmeadow, MA USA; 3 The Medicines Company, Parsippany, NJ USA; 4 Washington Hospital Center, Washington, DC USA

**Keywords:** Enterobacteriaceae, Costs, Readmission, Sepsis, Pneumonia, UTI

## Abstract

**Background:**

Enterobacteriaceae are common pathogens in pneumonia, sepsis and urinary tract infection (UTI). Though rare, carbapenem resistance (CRE) among these organisms complicates efforts to ensure adequate empiric antimicrobial therapy. In turn this negatively impacts such outcomes as mortality and hospital costs. We explored proportion of total costs represented by antibiotics, 30-day readmission rates, and per-day costs of inadequate antimicrobial coverage among patients with Enterobacteriaceae pneumonia, sepsis and/or UTI in the context of inappropriate (IET) vs. appropriate empiric (non-IET) therapy and carbapenem resistance (CRE) vs. susceptibility (CSE).

**Methods:**

We conducted a retrospective cohort study in the Premier Research database (2009–2013) of 175 US hospitals. We included all adult patients admitted with a culture-confirmed UTI, pneumonia, or sepsis as principal diagnosis, or as a secondary diagnosis in the setting of respiratory failure. Patients with hospital acquired infections or transfers from other acute facilities were excluded. IET was defined as failure to administer an antibiotic therapy in vitro active against the culture-confirmed pathogen within 2 days of admission.

**Results:**

Among 40,137 patients with Enterobacteriaceae infections (54.2% UTI), 4984 (13.2%) received IET. CRE (3.1%) was more frequent in patients given IET (13.0%) than non-IET (1.6%, *p* < 0.001). The proportions of total costs represented by antibiotics were similar in IET and non-IET (3.3% vs. 3.4%, *p* = 0.01), and higher among the group with CRE than CSE (4.2% vs. 3.4%, *p* < 0.001). The 30-day readmission rates were higher in both IET than non-IET (25.6% vs. 21.1%, *p* < 0.001) and CRE than CSE (29.7% vs. 21.5%, p < 0.001) groups. Each additional day of inadequate therapy cost an additional $766 (95% CI $661, $870, *p* < 0.001) relative to adequate treatment.

**Conclusions:**

In this large US cohort of Enterobacteriaceae infections, the cost of antibiotics was a small component of total costs, irrespective of whether empiric treatment was appropriate or whether a CRE was isolated. In contrast, each extra day of inadequate treatment added >$750 to hospital costs. Both CRE and IET were associated with an increased risk of readmission within 30 days.

**Electronic supplementary material:**

The online version of this article (10.1186/s13756-017-0286-9) contains supplementary material, which is available to authorized users.

## Background

Antimicrobial resistance remains a growing threat to public health and a vexing challenge to clinicians. Rates of in vitro susceptibility for most commonly utilized antibiotics continue to decline for both gram-positive and gram-negative organisms [1]. This is particularly problematic among such gram-negative pathogens as *Pseudomonas aeruginosa*, *Acinetobacter baumannii* and various Enterobacteriaceae [1–7]. Since prompt appropriate treatment is critical for treatment success, this rise in the risk of inappropriate empiric therapy (IET) associated with resistant organisms is a harbinger of potentially worse outcomes [8–17]. Exposure to IET is associated with longer durations of hospitalizations and greater healthcare costs, independent of its impact on mortality [18, 19]. Despite the link between inappropriate therapy and worsened outcomes, multiple obstacles preclude clinicians from effectively targeting these resistant organisms. These challenges include difficulty with risk stratification, concern about promoting further resistance through prescribing unnecessarily broad empiric coverage, and the acquisition costs of potentially active, newer antimicrobials. However, the trade-offs between these pathways have not been fully explored. For example, in a representative cohort of patients, on balance, does each day of exposure to inadequate antimicrobial treatment cost more than the potential savings from using less active but cheaper medications, which are more likely to be inadequate? Or what proportion of the overall hospital bill is attributable to antimicrobials and how, if at all, does it differ between patients given appropriate and inappropriate empiric treatment? Answering these questions may lend a broader perspective to the debate of risks and benefits of broad-spectrum treatment when warranted than simply focusing on acquisition costs.

Enterobacteriaceae represent frequent pathogens in multiple common infections such as urinary tract infection (UTI), sepsis and pneumonia. Not surprisingly, the rising prevalence of carbapenem resistant Enterobacteriaceae (CRE) heightens the risk for the clinician to prescribe IET, which, in turn, increases mortality [20]. The full economic impact of IET in this setting, however, is less well understood. Although in a prior study IET was associated with an approximately 5-day increase in length of stay (LOS) and a $10,000 increase in costs, other important economic outcomes have not been examined in this population [20]. Hence, we sought to explore the direct costs associated with antibiotics prescribed and also those attributable to delaying adequate treatment. We further examined rates of hospital readmission at 30-days in the setting of an index hospitalization with Enterobacteriaceae (both carbapenem susceptible [CSE] and CRE) in UTI, sepsis and/or pneumonia.

## Methods

We conducted a multi-center retrospective cohort study of patients admitted to the hospital with a UTI + sepsis (referred to throughout the paper as “UTI”), pneumonia and/or sepsis in the Premier Research database for the years 2009–2013. The aim of the current analysis was to quantify 30-day readmission rates, antibiotics cost as an absolute value and as a proportion of the total hospital costs, as well as the incremental daily contribution of delays in adequate antimicrobial treatment to increasing total hospital costs.

Because this study utilized an already existing HIPAA-compliant fully de-identified data, it was exempt from IRB review.

### Patient population

The current analysis was performed on a cohort previously described [20]. Briefly, patients were included if they were adults (age ≥ 18 years) hospitalized with Enterobacteriaceae UTI, pneumonia, and/or sepsis (cultured from a urinary, respiratory or blood source). UTI, pneumonia, and sepsis were identified via combinations of previously published ICD-9-CM codes [20–25]. In order to eliminate confounding cost calculations and isolate infection-related costs, only patients with community-onset (present on admission) infection were included. To differentiate infection from colonization, we further required subjects to be treated with an antibiotic beginning within the first two hospital days and continued for ≥3 consecutive days, or until discharge [22–24]. Patients were followed until death in or, if discharged alive from the hospital, for an additional 30 days for evidence of hospital readmission.

To establish the attributable per-day costs of inadequate antimicrobial coverage (defined as each day of not receiving an antimicrobial the pathogen is susceptible to), we analyzed a subgroup of the cohort who had the following characteristics: 1) They survived the hospitalization; 2) They, at some point during the hospitalization, received adequate coverage for their infection. Since our “appropriate/inappropriate” definition applies only to the empiric treatment period, we refer to “adequate/inadequate” treatment as a period that encompasses both, empiric and definitive time frames.

### Data source

Premier Research database, an electronic laboratory, pharmacy and billing data repository for years 2009 through 2013, contains ~15% of all hospitalizations nationwide. In addition to patient age, gender, race/ethnicity, principal and secondary diagnoses and procedures, the database contains a date-stamped log of all medications, laboratory tests, and diagnostic and therapeutic services charged to the patient or their insurer. We used data from 176 US institutions who submit microbiology data into the database. Eligible time began only following the commencement of microbiology data submission by each institution.

### Baseline variables

For a full description of baseline variable, please, refer to the previously published study [20]. Briefly, patient factors included demographic variables and comorbid conditions. The Charlson comorbidity index (CCI) score was computed as a measure of the burden of chronic illness, while ICU admission, mechanical ventilation and vasopressor use served as markers for disease severity. Hospital-level characteristics examined included geographic region, size, teaching status, and urbanicity.

### Microbiology and treatment variables and definitions

Urinary, blood and/or respiratory cultures had to be obtained within the first 2 days of hospitalization.

The following organisms were defined as Enterobacteriaceae of interest:

1. *Escherichia coli*
2. *Klebsiella pneumoniae*
3. Klebsiella oxytoca
4. *Enterobacter cloacae*
5. *Enterobacter aerogenes*
6. *Proteus mirabilis*
7. *Proteus spp.*
8. *Serratia marcescens*
9. *Citrobacter freundii*
10. *Morganella morganii*
11. *Providencia spp.*

CRE were defined as one of the above organisms where susceptibility testing yielded an “intermediate” or “resistant” result to at least one of the four carbapenems: imipenem, meropenem, ertapenem or doripenem.

IET was present if the antibiotic administered for the infection did not cover the organism based on reported in vitro information, or if appropriate coverage did not begin within 2 days of the positive culture being obtained.

The costs of antibiotics examined pertained to any antibiotics administered during the given hospitalization, regardless of whether they were used to treat the index infection or other infections.

### Statistical analyses

The complete details of statistical analyses of the cohort have been described previously [20]. For the current analyses, the following was also done.

To assign costs to the delay in appropriate empiric treatment, we categorized LOS into 3 groups: 1). number of days until the first index culture (the “pre” time), 2). number of days after the index culture until the first appropriate antibiotic (the period of interest), and 3). number of days after the first appropriate antibiotic until hospital discharge (the “post” time). It was important to adjust for the “pre” and “post” times so that the costs associated with these time periods were not attributed to the wrong period. The model structure was a generalized linear model with a logarithmic link to account for the skew in total costs (the outcome variable). In addition to the 3 time variables, other variables included all the other predictors known by hospital day 2 (i.e., all demographics, comorbidities, healthcare-associated (HCA) status, and a large number of treatments such as mechanical ventilation, vasopressor use, dialysis, inotropes, opioids, etc. as done in prior modeling [20]).

All inference tests were two-tailed, and a *p* value <0.05 was deemed a priori to represent statistical significance. All analyses were performed in Stata/MP 13.1 for Windows (StataCorp LP, College Station, TX).

## Results

Among 37,694 patients presenting to the hospital with a UTI, pneumonia or sepsis, who met the inclusion criteria and had treatment data, 4984 (13.2%) received IET. The prevalence of CRE was low. Specifically CRE accounted for 13.0% of cases within the IET cohort and 1.6% in non-IET groups (*p* < 0.001). Complete baseline, infection, treatment and hospital characteristics and outcomes of the entire population are available in an earlier publication and are reproduced in the Additional file 1: Table S1 (20). Briefly, with the exception of race (more likely black in the IET group than non-IET), and comorbidity burden (greater in IET than non-IET), other demographic variables were largely similar between the groups. Sepsis and UTI among those receiving IET were slightly less and pneumonia slightly more frequent upon admission. Except for the greater prevalence of mechanical ventilation among IET than the non-IET group (21.3% vs. 15.5%, *p* < 0.001), acute illness severity did not differ based on ICU admission or administration of vasopressors as a function of initial therapy appropriateness. Unadjusted hospital mortality was higher in patients receiving IET than non-IET (10.6% vs. 8.6%, *p* < 0.001) in both infection types. Both unadjusted LOS and costs were significantly higher in the IET group than in the group receiving non-IET. These relationships generally held irrespective of the infection type (Additional file 1: Table S1) (20).

The total median unadjusted antibiotics costs did not exceed $750 in any of the infection types, with the aggregated median antibiotic cost in the IET group higher than in the non-IET ($602, IQR [$230, $1422] vs. $441, IQR [$206, $919], *p* < 0.001) (Table 1). The highest proportion of the median total costs of hospitalization due to antibiotic costs reached 4.1% in the setting of appropriate treatment in pneumonia (Table 1). Across all infection types, though, the fraction of median hospital costs represented by antibiotic acquisition varied significantly between the IET and non-IET populations. Despite this statistical difference, the absolute difference in costs was trivial from a financial perspective. Specifically, for IET and non-IET patients, this proportion was 3.3% and 3.4%, respectively (Table 1).

**Table 1:** Unadjusted hospital resource utilization outcomes

	Non-IET		IET		
	*N* = 32,710		*N* = 4984		
Antibiotics costs, $	*Mean/Median*	*SD/IQR*	*Mean/Median*	*SD/IQR*	*P-value*
UTI					
Mean	779	1486	1333	2655	<0.001
Median	405	190, 844	586	226, 1332	<0.001
Sepsis					
Mean	958	2496	1736	3840	<0.001
Median	478	226, 1007	731	310, 1782	<0.001
Pneumonia					
Mean	887	1106	918	1202	<0.001
Median	552	269, 1067	459	183, 1148	0.098
Any					
Mean	845	1844	1381	2909	<0.001
Median	441	206, 919	602	230, 1422	<0.001
Antibiotics costs as a fraction of total hospital costs	*Mean/Median*	*SD/IQR*	*Mean/Median*	*SD/IQR*	*P-value*
UTI					
Mean	4.5%	4.4%	5.0%	5.2%	<0.001
Median	3.4%	1.9%, 5.8%	3.5%	1.8%, 6.4%	0.066
Sepsis					
Mean	4.3%	4.2%	4.3%	5.1%	0.923
Median	3.2%	1.8%, 5.5%	3.0%	1.4%, 5.3%	<0.001
Pneumonia					
Mean	5.3%	4.4%	4.6%	4.3%	<0.001
Median	4.1%	2.3%, 7.0%	3.2%	1.7%, 6.0%	<0.001
Any					
Mean	4.5%	4.4%	4.7%	5.0%	0.028
Median	3.4%	1.9%, 5.8%	3.3%	1.6%, 6.0%	0.012
30-day readmission	*Rate*	*N at risk*	*Rate*	*N at risk*	*P-value*
UTI	20.8%	16,028	24.8%	2194	<0.001
Sepsis	20.6%	9097	26.7%	1191	<0.001
Pneumonia	23.8%	3521	25.8%	294	0.204
Any	21.1%	28,646	25.6%	4279	<0.001

*IET* inappropriate empiric therapy, *SD* standard deviation, *UTI* urinary tract infection, *IQR* interquartile range

30-day readmission rates were high for all infection types (> 20%). More importantly, rates of readmission were significantly higher in those given IET compared to non-IET (25.6% vs. 21.1%, *p* < 0.001) (Table 1).

The sub-cohort of patients analyzed to assess the daily attributable cost of receiving inadequate antimicrobial coverage consisted of 27,953 patients who survived their index hospitalizations and at some point during their hospital stay received adequate treatment. The vast majority of them (78.5%) received such treatment as soon as infection was suspected and the culture obtained. In the remaining 21.5%, the mean (SD) duration of delay to appropriate therapy was 1.8 (1.1) days. In an adjusted analysis, each day’s delay in instituting adequate therapy added $766 (95% confidence interval $661, $870, *p* < 0.001) to the total cost of hospitalization. This represents 3.5% of the total mean daily hospital cost and is similar in magnitude to the actual direct costs for antibiotic acquisition.

## Discussion

In this study, we show that regardless of the appropriateness of initial antibiotic treatment, antibiotic costs represented less than 3.5% of the total hospital costs. We also demonstrate that the prevalence of 30-day readmission among the survivors of a hospitalization with an Enterobaceriaceae UTI, pneumonia and/or sepsis was over 20%, with those given initially inappropriate therapy facing a significantly higher risk for readmission.

Importantly, each additional day of inadequate therapy (among survivors) was associated with an appreciable cost of $766/day, and this cost became evident as soon as infection was suspected and culture obtained. Taken together, these results suggest that the additive costs of IET are high and independent of many confounders. Furthermore, there are hidden costs related to IET. Specifically, the attributable costs related to the delay in adequate therapy are similar in size to the costs for a day on a general medical floor in the US, while the increased rate of readmissions represents a potential for lost revenue to medical institutions, as the federal agencies may not reimburse for such readmissions. In other words, the total true costs of inappropriate therapy extend beyond the impact on the index event. In this sense our findings are novel in that they reveal that initial antibiotic treatment decisions have substantial downstream consequences that are important both for the patient and the healthcare institution.

To the best of our knowledge, this is the first study to address concerns around the costs of antibiotic therapy, including broad-spectrum agents available during the study timeframe, as empirical coverage for serious infections. The considerable expense associated with delay to adequate therapy may act to countervail fiscal concerns regarding the expenses associated with some of the newer therapies in appropriately targeted patients, particularly given that the median expenditures on antibiotics comprise less than 4% of the overall hospital costs. Put another way, acquisition costs must be viewed in the totality of the cost for hospitalization and potential readmission. The most expensive antibiotic may not be the one with the highest price but the one that is used as inappropriate initial therapy.

Under the best of all possible circumstances, a clinician would be able to order a bedside test to establish with a high degree of certainty, both the pathogen and its antimicrobial susceptibility profile before administering treatment. Unfortunately, such technologies remain only on the horizon, and even when available will raise concerns of overtreatment [26]. Many clinicians currently advocate for a probabilistic approach to risk stratification to guide the use of broad-spectrum antibiotic therapy [27]. The usefulness of such Bayesian approaches is limited in clinical practice for a pathogen like CRE, which, although rare (under 1.3% of all Enterobacteriaceae infections in the current study), raises the risk of IET significantly. In other words, a low pre-test probability (as displayed by the low prevalence of CRE) limits the conclusions that can be definitively drawn from such mathematical approaches. However, if a predictive test could identify with a high degree of confidence patients who do not require broad-spectrum coverage, its combination with molecular diagnostics could identify more precisely a more limited population that would require safeguarding with broader antimicrobials. Such bracketing of eligibility for newer agents along with the high cost of treating inadequately may both offset the concerns for draining the pharmacy budgets and improve patient outcomes. This hypothesis, however, requires exploration in future research.

Our novel result of the cost associated with each day’s delay to adequate coverage complements prior work. Zhang and coworkers in patients with sepsis recently reported that each hour’s delay in appropriate antimicrobial treatment is associated with a 0.1-day’s add-on to the post-infection onset hospital LOS [19]. While such an increment does not seem substantial, over days of delay this LOS increases dramatically. Our data build on this finding by calculating the actual costs associated with such delays and therefore serve to reinforce the point that the most expensive antibiotic is the one used inappropriately or for rescue therapy, irrespective of its acquisition cost.

Our cost calculation brings out the following important point: each additional day of inadequate therapy for an Enterobaceriaceae UTI, pneumonia or sepsis contributes as much to the total cost of the hospitalization as the total price of all antibiotics administered during the given hospitalization. Given the known improvement in the chances of survival with immediate appropriate treatment, this serves as further compelling evidence to start broadly and de-escalate as necessary [12, 14, 16].

Our study has a number of strengths and limitations. As a large multicenter cohort it is representative of US institutions, and thus has broad generalizability. Although susceptible to bias, particularly selection bias, we dealt with it by setting a priori enrollment criteria and definitions for the main exposures and outcomes. Though some misclassification is possible, particularly in the face of relying on administrative coding for case definition, the main exposure (IET) and outcomes (30-day readmission, antibiotics costs) are minimally susceptible to misclassification. At the same time, in at least some of the identified cases Enterobacteriaceae might have represented colonization, rather than a true infection. Finally, we did not examine how specific antibiotic regimens contribute to the costs of the hospitalization, so it remains unknown how much of those costs can be attributed to the newer more expensive agents. However, it may be assumed that those patients who received appropriate empiric treatment were more likely than those treated inappropriately to get the newer agents, particularly in the setting of CRE. At the same time, the time frame of the study predates coming to market of any of the newer broad-spectrum anti-gram-negative agents, and our analysis may need to be repeated when those data become available.

## Conclusions

Hospitalizations with Enterobacteriaceae are costly, and specific antibiotic agent choice exerts less impact on overall costs than antibiotic appropriateness. Given many lines of evidence that document that IET is detrimental to survival, it becomes a clinical imperative to adopt strategies and protocols that maximize rates of appropriate therapy. We demonstrate that concerns about the costs of broader-spectrum antibiotics, at least those available at the time of the analysis, appear unwarranted, since the total antimicrobial costs comprise only a modest proportion of total costs of hospitalization and must also be weighed against the potential for a no-pay event, such as a hospital readmission. Finally, the fact that each additional day of inadequate treatment is roughly equivalent in cost to the total per-patient cost of all antimicrobials administered is a reason to pause to reconsider these now clearer trade-offs in clinical decision-making. If other investigations confirm our findings, there may be a need for a paradigm shift to account for failure to cover serious infections appropriately.

## Additional file

Additional file 1: Table S1. Characteristics of the cohort based on the receipt of inappropriate empiric treatment. (DOCX 111 kb)

## Abbreviations

CCI: Charlson comorbidity index
CRE: Carbapenem resistant enterobacteriaceae
CSE: Carbapenem susceptible enterobacteriaceae
HCA: Healthcare-associated
HIPAA: Healthcare insurance portability and accountability act
ICD-9-CM: International classification of diseases, version 9, clinical modification
ICU: Intensive care unit
IET: Inappropriate empiric therapy
IQR: Interquartile range
IRB: Institutional review board
LOS: Length of stay
UTI: Urinary tract infection
