# Supplementary material for: 30-day readmission, antibiotics costs and costs of delay to adequate treatment of Enterobacteriaceae UTI, pneumonia, and sepsis: a retrospective cohort study
Source: Antimicrob Resist Infect Control. 2017 Dec 6;6:124. doi: 10.1186/s13756-017-0286-9 (PMC5717819; doi:10.1186/s13756-017-0286-9)
Supplement: Additional file 1: Table S1. — Characteristics of the cohort based on the receipt of inappropriate empiric treatment. (DOCX 111 kb) [file 13756_2017_286_MOESM1_ESM.docx]

**Supplemental Table 1. Characteristics of the cohort based on the receipt of inappropriate empiric treatment***

|  | **Non-IET** | **%** | **IET** | **%** | **P-value** |
| --- | --- | --- | --- | --- | --- |
|  | **N =32,710** | | **N = 4,984** | |  |
| ***Baseline characteristics*** |  |  |  |  |  |
| Mean age, years (SD) | 69.0 (16.0) |  | 69.4 (15.3) |  | 0.094 |
| Gender: male | 13,680 | 41.8% | 2,169 | 43.5% | 0.024 |
| Race |  |  |  |  |  |
| White | 23,921 | 73.1% | 3443 | 69.1% | <0.001 |
| Black | 4,384 | 13.4% | 862 | 17.3% |  |
| Hispanic | 919 | 2.8% | 163 | 3.3% |  |
| Other | 3,486 | 10.7% | 516 | 10.4% |  |
| Admission Source |  |  |  |  |  |
| Non-healthcare facility (including from home) | 21,450 | 65.6% | 3034 | 60.9% | <0.001 |
| Clinic | 1,093 | 3.3% | 138 | 2.8% |  |
| Transfer from ECF | 2,996 | 9.2% | 759 | 15.2% |  |
| Transfer from another non-acute care facility | 379 | 1.2% | 77 | 1.5% |  |
| Emergency Department | 6,688 | 20.4% | 959 | 19.2% |  |
| Other | 104 | 0.3% | 17 | 0.3% |  |
| Elixhauser Comorbidities |  |  |  |  |  |
| Congestive heart failure | 7,836 | 24.0% | 1509 | 30.3% | <0.001 |
| Valvular disease | 2,594 | 7.9% | 425 | 8.5% | 0.148 |
| Pulmonary circulation disease | 1,912 | 5.8% | 358 | 7.2% | <0.001 |
| Peripheral vascular disease | 3,564 | 10.9% | 577 | 11.6% | 0.152 |
| Paralysis | 3,289 | 10.1% | 770 | 15.4% | <0.001 |
| Other neurological disorders | 7,227 | 22.1% | 1269 | 25.5% | <0.001 |
| Chronic pulmonary disease | 9,079 | 27.8% | 1663 | 33.4% | <0.001 |
| Diabetes without chronic complications | 9,695 | 29.6% | 1623 | 32.6% | <0.001 |
| Diabetes with chronic complications | 3,152 | 9.6% | 524 | 10.5% | 0.052 |
| Hypothyroidism | 5,645 | 17.3% | 942 | 18.9% | 0.004 |
| Renal failure | 9,024 | 27.6% | 1540 | 30.9% | <0.001 |
| Liver disease | 1,774 | 5.4% | 245 | 4.9% | 0.138 |
| Peptic ulcer disease with bleeding | 15 | 0.0% | 2 | 0.0% | 1.000 |
| AIDS | 8 | 0.0% | 4 | 0.1% | 0.063 |
| Lymphoma | 508 | 1.6% | 74 | 1.5% | 0.716 |
| Metastatic cancer | 1,543 | 4.7% | 182 | 3.7% | 0.001 |
| Solid tumor without metastasis | 1,335 | 4.1% | 163 | 3.3% | 0.006 |
| Rheumatoid arthritis/collagen vascular | 1,422 | 4.3% | 215 | 4.3% | 0.914 |
| Coagulopathy | 4,626 | 14.1% | 540 | 10.8% | <0.001 |
| Obesity | 5,079 | 15.5% | 822 | 16.5% | 0.081 |
| Weight loss | 5,583 | 17.1% | 1117 | 22.4% | <0.001 |
| Fluid and electrolyte disorders | 17,961 | 54.9% | 2702 | 54.2% | 0.357 |
| Chronic blood loss anemia | 459 | 1.4% | 79 | 1.6% | 0.313 |
| Deficiency Anemia | 12,735 | 38.9% | 2096 | 42.1% | <0.001 |
| Alcohol abuse | 1,139 | 3.5% | 163 | 3.3% | 0.446 |
| Drug abuse | 789 | 2.4% | 103 | 2.1% | 0.135 |
| Psychosis | 1,979 | 6.1% | 294 | 5.9% | 0.676 |
| Depression | 4,859 | 14.9% | 806 | 16.2% | 0.018 |
| Hypertension | 20,987 | 64.2% | 3154 | 63.3% | 0.229 |
| Charlson Comoribidity Score |  |  |  |  |  |
| 0 | 10,353 | 31.7% | 1239 | 24.9% | <0.001 |
| 1 | 6,517 | 19.9% | 1072 | 21.5% |  |
| 2 | 6,595 | 20.2% | 1047 | 21.0% |  |
| 3 | 4,223 | 12.9% | 757 | 15.2% |  |
| 4 | 2,400 | 7.3% | 465 | 9.3% |  |
| 5+ | 2,622 | 8.0% | 404 | 8.1% |  |
| Mean (SD) | 1.9 (2.1) |  | 2.0 (2.0) |  | <0.001 |
| Median [IQR] | 1 [0, 3] |  | 2 [1 3] |  | <0.001 |
|  |  |  |  |  |  |
| ***Infection characteristics and treatment*** |  |  |  |  |  |
| Infection characteristics |  |  |  |  |  |
| Sepsis | 10,736 | 32.8% | 1468 | 29.5% | <0.001 |
| Pneumonia | 3,936 | 12.0% | 995 | 20.0% |  |
| UTI | 18,038 | 55.1% | 2521 | 50.6% |  |
| HCA | 11,413 | 34.9% | 2221 | 44.6% | <0.001 |
| CRE | 513 | 1.6% | 648 | 13.0% | <0.001 |
| Illness severity |  |  |  |  |  |
| ICU admission | 13,524 | 41.3% | 2074 | 41.6% | 0.720 |
| Mechanical ventilation | 5,064 | 15.5% | 1062 | 21.3% | <0.001 |
| Vasopressors | 4,929 | 15.1% | 709 | 14.2% | 0.111 |
| Antibiotics administered |  |  |  |  |  |
| Aminoglycosides | 3,694 | 11.3% | 351 | 7.0% | <0.001 |
| Antipseudomonal penicillins | 6,199 | 19.0% | 347 | 7.0% | <0.001 |
| Antipseudomonal floroquinolones | 15,995 | 48.9% | 2480 | 49.8% | 0.258 |
| Antipseudomonal penicillins with beta-lactamase inhibitors | 16,874 | 51.6% | 2008 | 40.3% | <0.001 |
| Extended spectrum cephalosporins | 12,174 | 37.2% | 1134 | 22.8% | <0.001 |
| Folate pathway inhibitors | 225 | 0.7% | 36 | 0.7% | 0.809 |
| Penicillins with beta-lacatamase inhibitors | 681 | 2.1% | 147 | 2.9% | 0.005 |
| Polymyxins | 102 | 0.3% | 32 | 0.6% | <0.001 |
| Tetracyclines | 210 | 0.6% | 15 | 0.3% | 0.004 |
| Tigecycline | 485 | 1.5% | 110 | 2.2% | <0.001 |
| Aztreonam | 1,319 | 4.0% | 258 | 5.2% | <0.001 |
|  |  |  |  |  |  |
| ***Hospital Characteristics*** |  |  |  |  |  |
| Area |  |  |  |  |  |
| Midwest | 8,848 | 27.0% | 1133 | 22.7% | <0.001 |
| Northeast | 4,397 | 13.4% | 950 | 19.1% |  |
| South | 13,579 | 41.5% | 1951 | 39.1% |  |
| West | 5,886 | 18.0% | 950 | 19.1% |  |
| Number of Beds |  |  |  |  |  |
| < 200 | 5,597 | 17.1% | 744 | 14.9% | <0.001 |
| 200 to 299 | 7,508 | 23.0% | 1171 | 23.5% |  |
| 300 to 499 | 10,540 | 32.2% | 1781 | 35.7% |  |
| 500+ | 9,065 | 27.7% | 1288 | 25.8% |  |
| Teaching | 12,096 | 37.0% | 1988 | 39.9% | 0.217 |
| Urban | 29,418 | 89.9% | 4574 | 91.8% | <0.001 |
|  |  |  |  |  |  |
| ***Hospital outcomes*** |  |  |  |  |  |
| Mortality | 3,234 | 9.9% | 607 | 12.2% | <0.001 |
| Mean (SD) LOS, days | 9.0 (8.5) |  | 14.7 (19.4) |  | <0.001 |
| Median [IQR] LOS, days | 7 [4, 11] |  | 9 [5, 16] |  | <0.001 |
| Mean (SD) costs, $ | 20,227 (25,616) |  | 33,216 (49,567) |  | <0.001 |
| Median [IQR] costs, $ | 12,719 [7,401, 23,275] |  | 17,386 [9,255, 35,625] |  | <0.001 |
| ***Hospital outcomes stratified by infection type*** | | |  |  |  |
| UTI |  |  |  |  |  |
| Mortality | 1,548 | 8.6% | 267 | 10.6% | <0.001 |
| Mean (SD) LOS, days | 8.5 (7.8) |  | 13.3 (17.1) |  | <0.001 |
| Median [IQR] LOS, days | 6 [4, 10] |  | 9 [5, 15] |  | <0.001 |
| Mean (SD) costs, $ | 18,103 (21,440) |  | 28,069 (40,490) |  | <0.001 |
| Median [IQR] costs, $ | 11,862 [7,015, 21,222] |  | 16,209 [8,828, 31,535] |  | <0.001 |
| Sepsis |  |  |  |  |  |
| Mortality | 1,356 | 12.6% | 260 | 17.7% | <0.001 |
| Mean (SD) LOS, days | 9.9 (9.9) |  | 18.9 (23.3) |  | <0.001 |
| Median [IQR] LOS, days | 7 [4, 12] |  | 12 [6, 22] |  | <0.001 |
| Mean (SD) costs, $ | 24,532 (32,043) |  | 47,881 (64,812) |  | <0.001 |
| Median [IQR] costs, $ | 15,048 [8,312, 28,558] |  | 25,121 [12,382, 55,529] |  | <0.001 |
| Pneumonia |  |  |  |  |  |
| Mortality | 330 | 8.4% | 80 | 8.0% | 0.726 |
| Mean (SD) LOS, days | 8.5 (7.6) |  | 12.0 (17.6) |  | <0.001 |
| Median [IQR] LOS, days | 7 [4, 10] |  | 7 [4, 13] |  | <0.001 |
| Mean (SD) costs, $ | 18,220 (21,710) |  | 24,623 (38,753) |  | <0.001 |
| Median [IQR] costs, $ | 11,742 [7,125, 20,561] |  | 13,040 [7,393, 26,339] |  | <0.001 |

*Reproduced from citation #20

IET = inappropriate empiric therapy; SD = standard deviation; ECF = extended care facility; AIDS = acquired immune deficiency syndrome; IQR = interquartile range; HCA = healthcare-associated; CSE = carbapenem sensitive Enterobacteriaceae; CRE = carbapenem resistant Enterobacteriaceae; UTI = urinary tract infection; ICU = intensive care unit; IQR = interquartile range 25-75%
